# The Lhermitte-Duclos disease: a rare bilateral cerebellar location of a rare pathology

**DOI:** 10.11604/pamj.2019.33.118.16809

**Published:** 2019-06-14

**Authors:** Mehdi Borni, Brahim Kammoun, Fatma Kolsi, Souhir Abdelmouleh, Mohamed Zaher Boudawara

**Affiliations:** 1 Department of Neurosurgery-UHC Habib Bourguiba, Sfax, Tunisia

**Keywords:** Dysplastic gangliocytoma, cowden's syndrome, gangliocytoma

## Abstract

Dysplastic gangliocytoma or Lhermitte-Duclos disease is a rare disorder characterized by a slowly progressive unilateral tumour mass of the cerebellar cortex. It is probably hamartomatous, although the exact pathogenesis remains unknown. Lhermitte-Duclos disease was recently encountered to be part of a multiple hamartoma-neoplasia complex (Cowden's syndrome). It typically presents in young adults, although it has been encountered at all ages. We present the case of bilateral cerebellar location of this pathology in a 50-year-old man presented with a progressive onset and worsening of headaches accompanied by nuchal rigidity, photophobia and nausea awakening each morning. Upon physical examination, the patient was awake with a discrete right vestibular syndrome made of positive Romberg without nystagmus. Magnetic Resonance Imaging (MRI) was performed and revealed salient “tiger stripe” appearance of the bilateral cerebellar cortex relevant to a Lhermitte-Duclos disease.

## Introduction

Dysplastic gangliocytoma or Lhermitte-Duclos disease is a rare disorder characterized by a slowly progressive unilateral tumour mass of the cerebellar cortex. It is probably hamartomatous, although the exact pathogenesis remains unknown. Lhermitte-Duclos disease was recently encountered to be part of a multiple hamartoma-neoplasia complex (Cowden's syndrome). It typically presents in young adults, although it has been encountered at all ages. Interestingly the genetics of childhood-onset appears different to the more common adult onset form.

## Patient and observation

**Figure 1:**
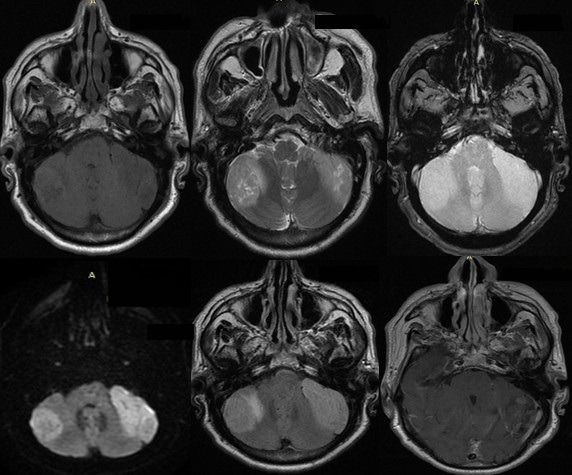
Cerebral MRI: two cerebellar lesions: iso signal weighted T1, hyper signal weighted T2, seat of small area of necrosis, heterogeneously enhanced, with hyper signal diffusion without decrease of the ADC and surrounded by oedema

It is about a 50-year-old man with no particular medical history who was admitted for a progressive onset and worsening of headaches over 4 months with by nuchal rigidity, photophobia and nausea awakening each morning without epilepsy or other localization signs. Upon physical examination, the patient was conscious with a discreet right vestibular syndrome made of positive Romberg without nystagmus or motor palsy. Cerebral MRI (Figure 1) was performed showing a first right cerebellar convexity lesion which was hyper intense on T2 weighted images containing small zones of necrosis in hyper intense T2 weighted signal and discrete hyper intense T1. This lesion is enhanced heterogeneously and moderately and surrounded by an edematous reaction. The second lesion was located in the other cerebellar lobe having the same signal as the right one and extending to the pontocerebellar angle displacing the acoustico-facial bundle and filling the pontocerebellar cistern pushing back the brainstem and without endo-canalar extension. The spectroscopy was performed in both of the 2 lesions revealing a choline peak and N-Acety Aspartate (NAA) drop.

## Discussion

The Lhermitte-Duclos disease, or dysplastic cerebellar gangliocytoma, is an unusual tumour arising from the cerebellar cortex. It most frequently presents in the third and fourth decades of life [1, 2] but the age at clinical manifestation ranges from birth [3] to the sixth decade [4]. There is no obvious sex preponderance. Regarding the nature of the disease, the original opinion expressed by Lhermitte and Duclos was that the tumour was a combination of a congenital malformation and a neoplasm arising from ganglion cells [5]. Other early observers of this entity suggested the lesion to be a “neurocystic blastoma” [2], “hamartoma” or “hyperplasia” [6]. Lhermitte and Duclos noted enlarged cerebellar folia containing circumscribed regions of abnormal ganglion cells [5]. They regarded the lesion as a combination of a ganglion cell neoplasm and a malformation originating from precursors of Purkinje cells. Bielschowsky and Simons suggested that the lesion is “an experiment of nature” derived from a local agenesis of the superficial cerebellar cortex, which resulted in a tumour composed of neuroblasts [2]. The histopathological differential diagnosis includes glioma, which might be confused with a very cellular dysplastic gangliocytoma and ganglion cell tumour [7]. The abnormal neurones found in Lhermitte-Duclos lesions are larger, rounder and more uniform in their morphological appearance than glioma cells. Neoplastic ganglion cells in gliomas diffusely infiltrate the gray matter, while in dysplastic gangliocytomas the abnormal ganglion cells are often clustered together. The association between Lhermitte-Duclos disease and other neoplasia is called Cowden syndrome which is an autosomal dominant hereditary cancer syndrome, characterized by mucocutaneous lesions and other systemic hamartomas associated with a high incidence of breast, thyroid and genitourinary malignancies [8]. Recently, thorough investigations support the contention that Lhermitte-Duclos disease and Cowden syndrome are part of a single spectrum best classified as a single phacomatosis [9]. Clinically, the duration of symptoms ranges from a few months to more than 10years [10]. Typically, the cause of clinical presentation is a posterior fossa mass lesion with headaches, cerebellar ataxia and visual disturbances and other cranial nerve palsies [1, 9]. Signs and symptoms of increased intracranial pressure, such as headaches, nausea and vomiting, papilloedema, mental disturbances and loss of consciousness occur in a later stage of the disease caused by the progressive mass effect of the growing tumour [1, 9]. MRI is certainly the imaging modality of choice today which shows a hypointense on T1-weighted images and show a very mild or no enhancement. The lack of contrast enhancement suggests insignificant disturbances of the blood-brain barrier and/or missing of extracellular oedema. On T2-weighted images the lesions present with a well circumscribed high signal intensity and an unique striated pattern with isointense bands within the area of hyper intensity, indicating the structures of widened gyri and consequently displaced sulci of the cerebellar cortex [11]. Surgery is definitely the therapeutic procedure commonly performed for the treatment [1, 9]. A sub-occipital approach is the surgical procedure generally performed [1, 9] and additional permanent ventricular shunt drainage occasionally preceded or followed tumor resection [9]. The absence of tumor limits in the depth of the cerebellar hemisphere constitutes the major technical problem during surgery. The radiation therapy should not be considered when in toto excision of the tumor and complete remission may be achieved [12].

## Conclusion

Dysplastic gangliocytoma of the cerebellum is of benign behaviour and its incidence is extremely rare. Its origin is still debatable. This lesion is hypo intense on T1- and hyper intense on T2-weighted MRI. Recognition of the disease is of particular importance, as the frequent but under-reported coexistence with Cowden syndrome, should prompt thorough clinical and apparative investigation to detect or exclude concomitant malignancies.

## Competing interests

The authors declare no competing interests.
